# Effect of Hyperthermic Intraperitoneal Perfusion Chemotherapy Combined with Radical Surgery and Capecitabine on Stage III Gallbladder Cancer

**DOI:** 10.1155/2021/4006786

**Published:** 2021-10-08

**Authors:** Sulai Liu, Zhendong Zhong, Weimin Yi, Zhangtao Yu, Zhihua Zhang, Guoyi Xia, Bo Jiang, Yinghui Song, Chuang Peng

**Affiliations:** ^1^Department of Hepatobiliary Surgery, Hunan Research Center of Biliary Disease, Hunan Provincial People's Hospital, The First Affiliated Hospital of Hunan Normal University, Changsha, Hunan Province, China; ^2^Biliary Disease Research Laboratory of Hunan Provincial People's Hospital, Key Laboratory of Hunan Normal University, Changsha, Hunan Province, China; ^3^Clinical Medical Technology Research Center of Hunan Provincial for Biliary Disease Prevention and Treatment, Changsha, Hunan Province, China; ^4^Department of Hepatobiliary Surgery, Changsha County People's Hospital, Hunan Provincial People's Hospital Xingsha Campus, Changsha, Hunan Province, China

## Abstract

**Purpose:**

The aim of the study was to investigate the effect of hyperthermic intraperitoneal perfusion chemotherapy (HIPEC) combined with radical surgery and capecitabine on stage III gallbladder cancer.

**Method:**

Seventy-eight patients with stage III gallbladder cancer treated in our hospital between December 2015 and April 2019 were retrospectively enrolled. Depending on the treatment approach, the patients were divided into the control group (radical surgery and capecitabine) and the HIPEC group (hyperthermic intraperitoneal perfusion chemotherapy combined with radical surgery and capecitabine). The patients were followed up by outpatient or through telephone until April 1, 2020. SPSS 19.0 software was applied for data analysis. Survival analysis was performed using the Kaplan–Meier method and parallel log-rank test.

**Results:**

There were 43 cases in the control group and 35 cases in the HIPEC group. There were no significant differences in operation time, lymph node metastasis, microvascular infiltration, and nerve invasion; there was no significant difference in postoperative complications between the two groups (*P* > 0.05). The average hospitalization time of the HIPEC group was 23.0 ± 6.9 days, which was longer than the 20.0 ± 5.8 days of the control group (*P* < 0.05). The body temperatures of HIPEC group patients at 0 h and 6 h after operation were higher than those of patients in the control group (*P* < 0.05); however, the body temperature of the two groups gradually became the same at 12–24 h after operation. There was no liver and kidney damage in the two groups after surgery. The platelets in the HIPEC group were less than those in the control group (*P* < 0.05). The median survival time of HIPEC was 19.2 months, which was longer than 15.3 months in the control group. The 1-year survival rates of the two groups were 91.43% vs. 76.71%, and the 2-year survival rates were 26.29% vs. 17.53%, respectively (*P* < 0.05).

**Conclusion:**

HIPEC combined with radical surgery and capecitabine for stage III gallbladder cancer can effectively prolong survival time without increasing surgery-related complications.

## 1. Introduction

Gallbladder carcinoma (GBC) is the most common type of biliary malignant tumor, and the main pathological type is adenocarcinoma [1]. Gallbladder cancer is characterized by local invasion and vascular invasion, and it is prone to extensive regional lymph node metastasis and distant metastasis. Most GBC is in the advanced stage when it is diagnosed, and the recurrence rate after the operation is extremely high [2]. Tumor recurrence is one of the main risk factors that affect the long-term survival time, including the direct dissemination of intraoperative tumors and the growth of micrometastases. The purpose of adjuvant treatment after GBC is to prolong the survival of patients.

Hyperthermic intraperitoneal chemotherapy (HIPEC), as an adjuvant treatment of abdominal malignant tumors, has achieved unique effects in the treatment of advanced gastric cancer peritoneal metastasis, colorectal cancer recurrence and metastasis, and primary peritoneal tumors. There have been more and more reports on the application of this treatment to hepatobiliary and pancreatic malignancies [3]. This study retrospectively analyzed seventy-eight patients with stage III gallbladder cancer treated in our hospital between December 2015 and April 2019. Clinical and pathological data were collected and explored to identify the effect of operation combined with hyperthermic intraperitoneal perfusion chemotherapy onstage III gallbladder cancer in order to provide a basis for the operation combined with hyperthermic intraperitoneal perfusion chemotherapy treatment of stage III gallbladder cancer.

## 2. Materials and Methods

### 2.1. Clinical Manifestations

The clinical data of 78 patients with stage III gallbladder cancer who underwent surgery from December 2015 to April 2019 in Hunan Provincial People's Hospital were collected.

### 2.2. Inclusion Criteria

1. Postoperative histopathological examination confirmed the patients with stage III gallbladder cancer
2. Completeness of cytoreduction (CCR) score was 0 to 1
3. Age varied from 18 to 75 years
4. No previous radiotherapy or chemotherapy
5. Preoperative liver function was in Child A
6. Kamofsky performance status (Kamofsky performance status, KPS) scoring standard was above 80 points

### 2.3. Exclusion Criteria

1. Severe cardiovascular and cerebrovascular diseases and liver and kidney insufficiency
2. Severe abdominal cavity adhesions
3. Concomitant with other types of malignant tumors
4. Blood coagulation insufficiency or platelets <100 × 10^12^/L
5. Long-term constipation or intestinal obstruction
6. Acute obstructive suppurative cholangitis or diffuse peritonitis
7. Lost to follow-up

### 2.4. HIPEC Treatment Termination Criteria

HIPEC treatment needs to be terminated that the patient cannot tolerate intraperitoneal hyperthermic perfusion chemotherapy. And those who did not complete the first hyperthermic perfusion chemotherapy were not included in the study.

### 2.5. Surgical Methods

Based on preoperative imaging, important organ functions, liver reserve functions, and resectability of the liver, surgical plans were planned to follow the NCCN guidelines: (1) routine liver S4b plus S5 resection was performed at stage T2 and T3; (2) right hepatectomy or enlarged right hepatectomy was performed for patients with liver bed involvement >2 cm, locating in the neck of the gallbladder, invading the gallbladder triangle, or involving with liver duodenal ligament lymph node metastasis; and (3) according to the results of lymph node biopsy in groups 13a and 16 during the operation, hepatoduodenal ligament lymph node dissection (groups 12 and 8) or enlarged lymph node (groups 12, 8, 9, and 13) dissection was selected. Cystic duct biopsy was routinely performed during the operation, and the positive patients needed to be combined with extrahepatic bile duct resection, ranging from the upper back of the pancreatic head to the first hepatic hilum, and a Roux-en-Y bile duct jejunum anastomosis [4].

### 2.6. HIPEC Methods

Before closing the abdomen, two tubes were placed under the left side of the diaphragm and the liver and kidney crypts as perfusion tubes respectively. Two drainage tubes in the lower abdomen were placed in the bilateral pelvic cavity as outflow tubes. Each drainage tube was placed at the level of the anterior axillary line. An external BR-TRG-II continuous intraperitoneal hyperthermic perfusion chemotherapy device was connected; the perfusion temperature was set to 43 ± 0.5°C; and the perfusion fluid was composed of saline (4,000 ml) and cisplatin (125 mg/m^2^). The perfusion speed was 400 ml/min. HIPEC lasted for 60 min each time [5–7]. The patients received intraperitoneal hyperthermic perfusion chemotherapy several times after the abdomen was closed, on day 2 and 4 after the operation.

### 2.7. Postoperative Adjuvant Treatment

Oral capecitabine (1,250 mg/m^2^) was given postoperatively twice a day on days 1 to 14 of a 3 week cycle for 24 weeks (eight cycles), and observation commenced within 16 weeks of surgery [8].

### 2.8. Follow-Up

The patients' gender, age, abdominal pain, bloating, jaundice, recurrence, survival status, etc. were followed up in outpatient and telephone visits after the operation. It is recommended that patients should be reviewed every month for the first six months after the operation, every three months after six months later, and every six months after one year later. Recurrence was defined as both the recurrence of jaundice and new lesions on imaging. The endpoint of follow-up was the death of patients or the follow-up time until April 1, 2020.

### 2.9. Statistical Analysis

SPSS 19.0 software was applied for data analysis. Categorical data were compared using frequencies expressed as percentages and compared with chi-squared testing. Operation time, hospitalization days, anal exhaust time, body temperature changes, postoperative liver, and kidney function, platelets, and so on were analyzed by variance among subgroups. Overall survival analysis was performed using the Kaplan–Meier method and parallel log-rank test. Prognostic factors were analyzed by univariate analysis and multiple Cox regression model. *P* < 0.05 was considered statistically significant.

## 3. Results

### 3.1. Clinicopathological Parameters of GBC Patients

According to the inclusion and exclusion criteria, a total of 78 patients met the requirements. Forty-three patients in the control group received surgery and capecitabine treatment, including 14 males and 29 females. The age distribution ranges from 35 to 74 years old. Thirty-five patients in the HIPEC group underwent surgery, HIPEC, and capecitabine treatment, 11 males and 24 females, aged between 43 and 74 years old, and received HIPEC treatment 2.1 times per capita (see Table 1 for general information). The hospital stay in the HIPEC group was longer than that in the control group (*P*=0.045).

### 3.2. Effect of Hyperthermic Perfusion Chemotherapy on Body Temperature

**Figure 1:**
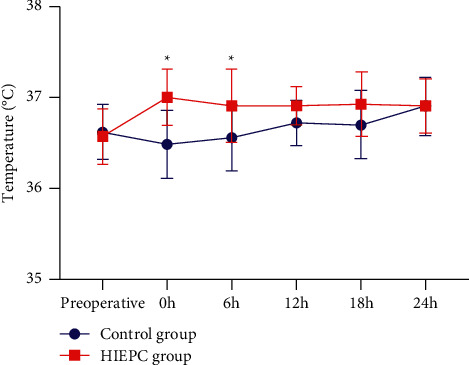
Changes in body temperature of the two groups of patients: temperature measurements at 0 h and 6 h after the operation showed that the body temperature of the HIPEC group was higher than that of the control group (*P* < 0.05). There was no difference in body temperature between the two groups at 12, 18, and 24 hours after operation (*P* > 0.05).

There was no difference in the mean body temperature at the beginning of the operation (preoperation) between the two groups. 1 patient in the HIPEC group had a low fever before the operation, and 2 patients in the control group had a low fever before the operation. Temperature measurements at 0 h and 6 h after operation showed that the body temperature of the HIPEC group was higher than that of the control group (*P* < 0.05). After 6 hours of operation, the body temperature gradually became similar for two groups. And there was no difference in body temperature between the two groups at 12, 18, and 24 hours after the operation (*P* > 0.05; see Figure 1 for details).

### 3.3. Effect of Hyperthermic Perfusion Chemotherapy on Postoperative Complications

There was no difference in liver function and kidney function between the two groups. 2 patients had tube blockage during the second time's treatment of HIPEC. After flushing and retreating the tube, the treatment was successfully completed, but the third treatment was not performed. There was no patient out of the tube. Also, there was no difference in the time of anal exhaust after the operation. The overall infection rate was 30% (13/43) in the control group and 34% (12/35) in the HIPEC group (*P*=0.703). There was no postoperative bleeding or anastomotic leakage that occurred in the two groups (see Table 2 for details).

### 3.4. Survival Analysis

**Figure 2:**
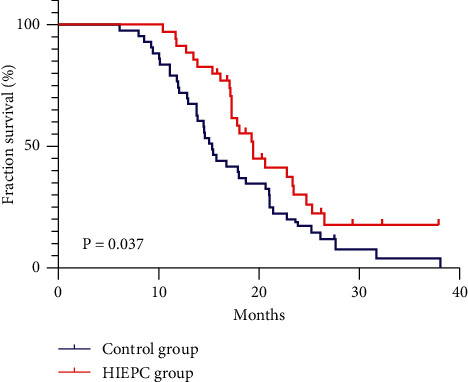
Cumulative survival of patients with control group patients and HIPEC group patients was determined by the Kaplan–Meier method. The median survival of the surgery combined with the gemcitabine treatment group in this study was 15.3 months. And the median survival time of patients treated with HIPEC was 19.2 months (*P*=0.037).

Sixty-five cases (83.3%) were followed up to the endpoint; there were no perioperative deaths; the average follow-up time of these patients was 20.4 ± 3.2 months. The median survival of the surgery combined with the gemcitabine treatment group in this study was 15.3 months. On this basis, the median survival time of patients treated with HIPEC was 19.2 months (*P*=0.037). The 1-year survival rates of the two groups were 91.43% vs. 76.71% and the 2-year survival rates were 26.29% vs. 17.53%, respectively (see Figure 2 for details). Univariate Cox proportional hazard regression analyses showed that lymph node metastasis, nerve invasion, and treatment approach were prognostic factors of stage III GBC patients (*P* < 0.05). Including lymph node metastasis, nerve invasion, and treatment approach into the COX model and performing multivariate analysis showed that lymph node metastasis (OR = 1.813; 95% confidence interval: 1.094–3.005; *P*=0.24), nerve invasion (OR = 1.801; 95% confidence interval: 1.082–2.998; *P*=0.26), and treatment approach (OR = 0.534; 95% confidence interval: 0.320–0.892; *P*=0.38) were independent factors affecting prognosis (see Table 3 for details).

## 4. Discussion

Gallbladder cancer is a common malignant tumor of the biliary tract. Radical surgical resection is the only method that is expected to cure gallbladder cancer clinically. However, the surgical resection rate is low, and the prognosis is extremely poor because the disease is often diagnosed in the middle and advanced stages [1]. Adenocarcinoma is the most common pathological type, which is not sensitive to radiotherapy or chemotherapy. Thus, the effect of adjuvant treatment is still controversial. The current chemotherapeutic drugs used in gallbladder cancer include platinum, gemcitabine, fluorouracil, etc. [9, 10]. HIPEC has achieved unique effects in the treatment of peritoneal cancer (PC). In addition, HIPEC is also used in advanced diseases such as gastric cancer, colorectal cancer, ovarian cancer, and endometrial cancer. Moreover, it is also reported that cytoreductive surgery (CRS) combined with HIPEC was applied for the treatment of advanced gallbladder cancer. However, their studies only had a very limited number of patients [11, 12]. In this study, seventy-eight patients with stage III gallbladder cancer treated with operation and capecitabine or HIPEC combined operation and capecitabine were enrolled to identify the effect of HIPEC onstage III gallbladder cancer.

When considering the implementation of HIPEC after the operation, the inclusion and exclusion criteria should be formulated under the premise of ensuring safety. The main contraindications of HIPEC treatment include severe abdominal cavity adhesion, intestinal obstruction, anastomotic edema, severe bleeding tendency, obvious liver and kidney insufficiency, severe cardiovascular and cerebrovascular system diseases, etc. Meanwhile, the surgical treatment of advanced gallbladder cancer is relatively complicated for requiring combined partial liver resection and lymph node dissection. In patients with gallbladder cancer undergoing partial hepatectomy combined with HIPEC, Child A liver function is required [3]. According to the inclusion and exclusion criteria, finally, 43 patients were enrolled in the control group, and 35 patients were in the HIPEC group.

Among the 78 patients, female patients are the majority, and 62.8% of patients have gallbladder stones, which is basically in line with other studies [13]. There was a difference in the number of days of hospitalization between the two groups. The patients in the HIPEC group had longer hospitalization time for patients needed to extend the extubation time of the abdominal drainage tube. Moreover, there were six patients who had grade III and IV complications. In order to reduce the hospitalization time, HIPEC patients may benefit from enhanced monitoring.

Due to the complexity of gallbladder cancer surgery, the general operation time is long, so intraoperative hypothermia is prone to occur. Perioperative hypothermia has many adverse effects on the body. A large number of evidences have shown that even if mild hypothermia occurs during or after surgery, it can also lead to many undesirable consequences [14]. Severe hypothermia can also cause internal environment disorders, abnormal coagulation function, immune function damage, respiratory depression, etc. which directly threatens the treatment effect and life safety of patients [15]. In this study, the preoperative body temperature of the two groups of patients was similar, and the temperature after returning to the ward showed that the body temperature of the control group was lower than that of the HIPEC group. Compared with the control group, HIPEC has corrected the hypothermia caused by long-term surgery to a certain extent and promoted body temperature recovery. After 6 hours after the operation, the body temperature of the two groups gradually recovered to a similar level, and HIPEC did not increase postoperative complications such as infection and fever. Cisplatin was applied to HIPEC did not cause significant liver and kidney damage, and only one patient with myelosuppression was corrected after symptomatic treatment.

In this study, the most common complication of HIPEC treatment was gastrointestinal reactions, which were manifested as the discomfort of the abdomen and delayed anus and defecation. After HIPEC treatment, there were no serious surgical-related complications such as hepatic wound bleeding, bile leakage, and anastomotic leakage. Also, there was no difference in the incidence of postoperative complications between the two groups. It is reported that CRS-HIPEC is associated with improved cancer survival but an increased risk of infection in one hundred patients predominantly for colorectal cancer and pseudomyxoma peritonei. The overall infection rate is 43%, and the most common site of infection is surgical site infection accounting for 27% [16]. Another study pointed that the overall complications were observed in 82 of 155 patients with peritoneal carcinomatosis who underwent CRS and HIPEC procedures. Infectious complications were the most important cause of perioperative morbidity and death in CRS and HIPEC. The surgeon-/center-related factors play an important role in infectious morbidity as well as patient and tumor characteristics [17]. In this study, the overall infection rate is 30% (13/43) in the control group and 34% (12/35) in the HIPEC group. The incidence of postoperative infection is lower than that of peritoneal cancer surgery or colon cancer surgery considering that the operation of gallbladder cancer does not involve gastrointestinal operation. However, there was no difference between the control group and the HIPEC group. This suggested HIPEC treatment did not increase the incidence of surgical-related infections.

There is little clinical data on the survival of patients with stage III gallbladder cancer. One research stated that the 5-year survival rates were 8% for stage IIIa and 7% for stage IIIb [18]. Another study showed that the median survival time of patients with advanced GBC was less than 1 year [19]. In the results of a study of advanced cholangiocarcinoma with the value of adjuvant chemotherapy after surgery, it was found that the median overall survival of stage III cholangiocarcinoma was about 20 months [20]. The median survival of the surgery combined with the gemcitabine treatment group in this study was 15.3 months. On this basis, the median survival time of patients treated with HIPEC was 19.2 months, suggesting that the survival time of patients after stage III surgery in our center is similar to the data of many medical centers [21, 22], and HIPEC could prolong the median survival time of patients (*P*=0.037). The 1-year survival rates of the 2 groups were 91.43% vs. 76.71%, and the 2-year survival rates were 26.29% vs. 17.53%, respectively. It is well known that GBC patients with lymph node metastasis and nerve invasion have a more poor prognosis than those without [23, 24]. Also, in this study, we found the same conclusion. Moreover, multivariate analysis showed that the treatment approach was one of the independent factors affecting prognosis. This indicated that HIPEC combined with radical surgery and capecitabine on stage III gallbladder cancer could increase survival benefits.

In short, radical surgery combined with postoperative capecitabine chemotherapy is one of the standard treatments for stage III gallbladder cancer. On this basis, combined with HIPEC can effectively prolong survival time without increasing surgery-related complications. This study provides a basis for the new comprehensive treatment of stage III gallbladder cancer.

## Figures and Tables

**Table 1:** Clinicopathological parameters of GBC patients.

Clinical features	Control group (*N* = 43)		HIPEC group (*N* = 35)		*P* value
	N	%	N	%	
Age (years)					0.666
≤60	20	46.51	18	51.43	
>60	23	53.49	17	48.57	
Gender					0.915
Female	29	67.44	24	68.57	
Male	14	32.56	11	31.43	
Stones					0.222
Negative	15	34.88	17	48.57	
Positive	28	65.12	18	51.43	
Tumor location					0.967
Bottom and body of gallbladder	26	60.47	21	60.00	
Gallbladder neck and cystic duct	17	39.53	14	40.00	
CEA					0.177
Normal	28	65.12	24	68.57	
High	15	33.88	11	31.43	
CA19-9					0.156
Normal	24	55.81	25	71.43	
High	19	44.19	10	28.57	
CA125					0.872
Normal	29	67.44	23	65.71	
High	14	32.36	12	34.29	
CA242					0.995
Normal	27	62.79	22	62.86	
High	16	37.21	13	37.14	
Lymph node metastasis					0.906
Negative	24	55.81	20	57.14	
Positive	19	44.19	15	42.86	
Microvascular infiltration					0.369
Negative	32	74.42	29	82.86	
Positive	11	25.58	6	17.14	
Nerve invasion					0.956
Negative	28	65.12	23	65.71	
Positive	15	34.88	12	34.29	
Body surface area (m^2^)	1.54 ± 0.49		1.58 ± 0.35		0.492
Tumor size (cm)	3.6 ± 1.6		4.1 ± 1.4		0.109
Operation time (minutes)	335 ± 103		350 ± 65		0.586
Hospital stay (days)	20.0 ± 5.8		23.0 ± 6.9		0.029∗

Note: *χ*2 test was used to compare the distribution of clinical features between control group patients and HIPEC group patients. CA19-9 normal reference range: 0–35 U/mL. CA242 normal reference range: 0–20 U/mL. CEA normal reference range: 0–5 ng/mL. CA125 normal reference range: 0–35 U/mL. ^*∗*^*P* value < 0.05 was considered significant.

**Table 2:** Postoperative index of GBC patients.

Postoperative index	Control group	HIPEC group	*P* value
ALT (U/L)	46.3 ± 26.7	46.8 ± 27.6	0.933
AST (U/L)	38.0 ± 19.1	35.8 ± 18.4	0.624
GGT (U/L)	105.8 ± 71.1	83.4 ± 39.1	0.098
Cr (*μ*mol/L)	49.5 ± 10.7	53.9 ± 12.6	0.103
BUN (mmol/L)	3.4 ± 1.5	3.5 ± 1.0	0.926
PLT (x10^9/L)	296.7 ± 75.8	233.1 ± 79.0	0.001^∗^
Anal exhaust time (days)	4.1 ± 1.3	4.7 ± 1.5	0.324
Infection	13/43	12/35	0.703
Clavien–Dindo classification (I/II)	12/43	8/35	0.654
Clavien–Dindo classification (III/IV)	6/43	3/35	0.459

ALT: alanine transferase; AST: aspartate aminotransferase; GGT: *γ*-glutamyl transpeptidase; Cr: creatinine; BUN: urea nitrogen; PLT: platelets.

**Table 3:** Multivariate analysis of factors contributing to overall survival in 78 GBC patients.

Variables	Univariate analysis		Multivariate analysis	
	HR (95% CI)	*P* value	HR (95% CI)	*P* value
Age (≤60 vs. >60)	1.514 (0.923–2.483)	0.100	—	—
Gender (female vs. male)	1.309 (0.733–2.337)	0.363	—	—
Stones (negative vs. positive)	1.336 (0.801–2.229)	0.268	—	—
CEA (≤5 ng/mL vs. >5 ng/mL)	1.341 (0.699–2.573)	0.378	—	—
CA19-9 (≤35 U/L vs. >35 U/L)	0.793 (0.485–1.298)	0.357	—	—
CA125 (≤35 U/L vs. >35 U/L)	1.353 (0.806–2.274)	0.253	—	—
CA242 (≤20 U/L vs. >20 U/L)	0.923 (0.543–1.569)	0.768	—	—
Microvascular infiltration (negative vs. positive)	1.414 (0.786–2.542)	0.248	—	—
Lymph node metastasis (negative vs. positive)	1.841 (1.108–3.058)	0.019	1.813 (1.094–3.005)	0.024
Nerve invasion (negative vs. positive)	2.003 (1.188–3.378)	0.011	1.801 (1.082–2.998)	0.026
Treatment approach (control vs. HIPEC)	0.578 (0.354–0.971)	0.037	0.534 (0.320–0.892)	0.042

Note: univariate and multivariate analysis of prognostic factors in 78 GBC patients was included in the survival analysis. Statistical analyses were performed by Cox proportional hazards regression. A *P* value < 0.05 was considered significant. CI, confidence interval.

## Data Availability

Data and materials are included within the manuscript.
